# Sufficiency of current practice: How well does the Strengths and Difficulties Questionnaire detect clinically elevated posttraumatic stress, anxiety, and depression symptoms in children in care?

**DOI:** 10.1002/jcv2.70058

**Published:** 2025-10-30

**Authors:** Eva A. Sprecher, Katherine Shelton, Lisa Holmes, Bethan Carter, Charlotte Robinson, Maryam Javed, Ruby Khan, John MacLeod, Jeongeun Park, Julie Selwyn, Iram Siraj, Ching So, Rachel M. Hiller

**Affiliations:** ^1^ Research Department of Clinical Educational and Health Psychology University College London (UCL) London UK; ^2^ School of Psychology Cardiff University Wales UK; ^3^ School of Education and Social Work University of Sussex Brighton UK; ^4^ Medical School University of Bristol Bristol UK; ^5^ Department of Education University of Oxford Oxford UK; ^6^ Froebel Department of Primary and Early Childhood Education Maynooth University Maynooth Ireland

**Keywords:** anxiety, children in care, depression, mental health assessment, PTSD, screening questionnaires

## Abstract

**Background:**

It is well‐established that children living in care are at far greater risk of mental health difficulties than their peers. This includes common and trauma‐specific mental health conditions, including depression, anxiety and posttraumatic stress disorder (PTSD). In England, the mental health of children in care is monitored using the caregiver‐report Strengths and Difficulties Questionnaire (SDQ). Our aim was to understand the sufficiency of current screening practices for children in care. We investigated how sensitive the SDQ was to clinically elevated PTSD, anxiety, and depression symptoms.

**Methods:**

The sample included 491 children aged 10–18 years old, under local authority care, and their caregiver where available (*n* = 342 carers, including foster carers, kinship carers and residential keyworkers). Children and caregivers completed the SDQ, and children also completed standardised measures of anxiety and depression and PTSD symptom, using established cut‐offs for clinically elevated symptoms.

**Results:**

Most of the sample scored above clinical threshold on at least one measure. Caregiver‐reported SDQ scores were weakly correlated with child self‐reported PTSD, anxiety, and depression scores, while self‐reported SDQ scores were moderately correlated with their self‐report on the other symptom scales. A large proportion of children scoring in the clinical range on the anxiety (44%), depression (46%), or PTSD (48%) symptoms measures did not score in the carer‐report SDQ clinical range. Similar patterns were found using the self‐report SDQ, with somewhat higher detection rates found when combining self‐ and carer‐report SDQ.

**Conclusion:**

Relying only on the carer‐report SDQ as a mental health screening tool is likely inadequate to detect mental health symptomology in children in care. Whilst this was never the purpose of the SDQ, it is how it is commonly used in practice. We discuss the benefits of including children's voices and disorder‐specific screening tools (e.g., for PTSD) into mental health assessments for children living in care.

## Key Points

**What's Known**

- Children living in care are at much greater risk of mental health difficulties than peers, including anxiety, depression and posttraumatic stress disorder (PTSD).
- Current screening of mental health difficulties for children in England largely relies on the carer‐report Strengths and Difficulties Questionnaire (SDQ).

**What's New**

- The carer‐report SDQ misses over 4 in 10 children in care with elevated posttraumatic stress, anxiety or depression symptoms based on standardised symptom screening tools.

**What's Relevant**

- Current, often sole, reliance on the carer‐reported SDQ to screen for the mental health difficulties of children in care is not sufficient for identifying need.
- Integrating child self‐report and disorder‐specific symptom measures are important steps for improving current mental health screening practices for children in care.

## INTRODUCTION

There are currently over 100,000 children under local authority care across the UK, living with foster carers, in residential children's homes, or in other settings (Department for Education, 2024a, 2024b; Senedd Research, Welsh Government, 2024; Northern Ireland Executive, 2025; Scottish Government, 2025). Local authority care, internationally often called ‘state’ or ‘out‐of‐home’ care, is defined as children being taken into care on behalf of the UK state, either resulting from a court order or voluntary agreement from a child's parents. Most children enter care due to experiencing substantial early adversity in their family home including abuse, neglect, witnessing domestic violence, and/or parental mental illness or substance addiction (Department for Education, 2024a; Hiller et al., 2021). Once in care, many face ongoing instability, including placement (and thus caregiver) changes and separation from siblings. Such experiences can have a profound influence on mental health, with epidemiological research showing children in care are five times more likely to meet diagnostic criteria for a mental health condition than peers with no experience of care (Ford et al., 2007). This elevated risk persists across specific disorders, with estimates suggesting children in care are twice as likely to meet criteria for an anxiety disorder or depression, and 12 times as likely to meet diagnostic criteria for post‐traumatic stress disorder (PTSD) (Coughlan et al., 2024; Ford et al., 2007). The heightened risk of psychological distress amongst children in care has led to calls for improved screening and early identification of their mental health needs (Power et al., 2024).

Presently, across the UK there is no standard approach for screening of mental health difficulties for children in care. In England specifically, all local authorities are required to obtain yearly carer‐report Strengths and Difficulties Questionnaire (SDQ) (Goodman, 1997) for all children 4–16 years old in their care who have been looked after for more than 12‐month (Department for Education, 2024b). This is part of mandatory reporting of total care‐report SDQ scores to the Department for Education (Cocker et al., 2018; Department for Education, 2024b; UK Parliament, 2016). This reporting relies on carer‐report SDQ, rather than child‐report, with national reporting of proportions of children falling into ‘borderline’ or ‘abnormal’ clinical ranges. Within local authorities, this screening tool is also often used to identify children in need of support, again using the measures established ‘borderline’ or ‘abnormal’ cut‐offs to trigger further assessment, potential intervention or service access. Across Wales, Northern Ireland and Scotland there is no statutory requirement for local authorities reporting of mental health or wellbeing data relating to children living in care. The SDQ provides an important opportunity for screening the mental health needs of children living in care and identifying those who may benefit from further assessment or support.

In the UK context, mental health service provision for children in care can differ substantially between regions, and may include NHS child and adolescent mental health services (CAMHS), but could also be a local authority, voluntary sector, or joint commission service (McGuire et al., 2025). Beyond children's social care, the caregiver‐ and teacher‐report SDQs are also one of the most widely used routine outcome measures within CAMHS (Johnston & Gowers, 2005), with increasing suggestion of its use as a tool for screening or assessing new referrals (Mathai et al., 2002). Given the wide use of the SDQ for children in care and potential implications for support, it is essential to fully understand the sufficiency of these established methods.

Research has established the validity and reliability of the SDQ amongst samples of children in care for identifying general externalising and internalising difficulties (Goodman et al., 2004; Marquis & Flynn, 2009), but there are concerns about relying on carer‐report SDQ alone as a screening tool (Wright et al., 2019). Amongst the general population and at‐risk samples, limitations of the SDQ in screening for disorder‐specific symptomology have been identified (Brøndbo et al., 2011; Goodman et al., 2000; Stolk et al., 2017). There is mixed evidence regarding the SDQ's ability to detect depression or anxiety‐specific symptomology. Work with community samples (Armitage et al., 2023) and using multi‐informant ratings (Goodman et al., 2000) has shown good specificity of the SDQ for detecting anxiety and depression in children. However, research with clinical populations of young people suggest weaknesses in the SDQ's detection of patients requiring further psychiatric evaluation (Brøndbo et al., 2011). There also is wide acknowledgement that the SDQ has limited sensitivity in detecting the psychological sequalae of trauma such as PTSD symptoms (Stolk et al., 2017; Tarren‐Sweeney et al., 2019). These studies have not been replicated amongst children in care in the UK, with no studies investigating the sufficiency of current statutory practice, which rely on the carer‐report SDQ for identifying mental health needs in this group. Understanding its sufficiency related to disorder‐specific symptom screeners is particularly important due to increasing use of these standardised screening tools instead of diagnostic assessments tools in UK child mental health services (McGuire et al., 2025; Sayal et al., 2025).

### Aims

This study aimed to explore the sufficiency of the SDQ as a mental health screening tool for PTSD, anxiety and depression symptomology amongst children living in care in England and Wales. Specifically:

1. Are there significant associations between self‐ and carer‐report SDQ total and emotional difficulties scores, and self‐report scores on disorder‐specific symptom screening tools for PTSD (CRIES‐8), anxiety and depression (RCADS‐25)?
2. To what extent are children with above‐threshold symptoms on disorder‐specific self‐report screening tools for PTSD, anxiety and depression missed by a reliance on either exclusive use of self‐ or carer‐report SDQ total score or emotional sub‐scale thresholds, and which children are more likely to be missed by such cut‐offs?

## MATERIALS AND METHODS

This study analysed data from the baseline assessments from two studies: the CCAT‐S study (Hiller et al., 2021) (*n* = 100) where recruitment occurred between 2016 and 2018 and the ReThink Programme (Hiller et al., 2023) (*n* = 391) where recruitment occurred between 2022 and 2024. Both samples were children under local authority care and used common measures.

### Participants

Participants were recruited from 15 local authorities across England (*n* = 12; 86% of participants) and Wales (*n* = 3; 14% of participants). Participants were 491 children living in local authority care, aged 10–18 years old. Primary caregivers were also invited to participate, from which there were a total of 342 carers (foster carers, kinship carers or keyworkers for those in residential care). Inclusion criteria were intentionally broad, with exclusion criteria only for severe current active suicidality or psychosis, or where English or intellectual ability (moderate to severe intellectual impairment) meant children would have been unable to complete questionnaires. The demographic characteristics of the sample are presented in Table 1.

**TABLE 1:** Description of study sample demographic characteristics.

	Children (*n* = 491)	Caregivers (*n* = 342)
	*M* (SD)	*M* (SD)
Child age	13.57 (2.50)	‐
Carer age	52.41 (10.04)	‐
Placement length	3.92 (3.21)^a^	‐

	*N* (%)	*N* (%)
Child gender		
Boy	238 (48.5)	‐
Girl	235 (47.9)	‐
Non‐binary	10 (2.0)	‐
Prefer not to say	4 (0.8)	‐
Not known	4 (0.8)	‐
Child sex at birth		
Female	249 (50.7)	‐
Male	242 (49.3)	‐
Carer gender		
Man	‐	40 (11.7)
Woman	‐	300 (87.7)
Not known	‐	2 (0.6)
Child ethnicity		
Asian	10 (2.0)	
Black	39 (7.9)	‐
Mixed	54 (11.0)	‐
Other	11 (2.2)	‐
White	367 (74.7)	‐
Not known	10 (2.0)	‐
Carer ethnicity		
Asian	‐	3 (0.9)
Black	‐	21 (6.1)
Mixed	‐	4 (1.2)
Other	‐	2 (0.6)
White	‐	310 (90.6)
Not known	‐	2 (0.6)
Placement type		
Foster care	370 (75.4)	276 (80.7)
Kinship care	54 (11.0)	42 (12.3)
Residential/semi‐independent/supported	50 (10.2)	21 (6.1)
Other	11 (2.2)	1 (0.3)
Not known	6 (1.2)	2 (0.6)
Country		
England	422 (85.9)	294 (86.0)
Wales	69 (14.1)	48 (14.0)

^a^ As placement length was based on caregiver‐report this value is only for *n* = 342 children where carer data was available.

Almost 30% of children (*n* = 149 of 491) did not have a corresponding caregiver report completed. This was predominantly because an appropriate caregiver could not be identified by researchers or children, caregivers were too busy to complete measures, or caregivers did not consent to participate. These children were more likely to be living in non‐familial (residential, semi‐independent or independent) placements (*p <* .001), have non‐White ethnicities (*p* = .010) and be older (*p <* .001), but no sex or placement duration differences were found (see Table S1 in Supporting Information S1).

### Ethics

Projects received ethical approval from University of Bath (Ref 16/IEC08/0025) and UCL (Ref 22253/001) Research Ethics Committees. Local approval was gained from participating local authorities and national approval was gained for the ReThink Programme (due to project scale) from the Association of Directors of Children's Services. Written consent was provided by the local authority (who hold parental responsibility), with assent provided by the child (or consent if 16+ years old). Caregivers provided their own written consent.

### Procedure

A total of 3177 children were consented by local authorities to take part in this research (CCAT‐S: *n* = 242; ReThink: *n* = 2935). Of these children, only 2113 were contactable and eligible for this research. Of eligible and contactable children, 25.7% consented or assented to take part in this research (*n* = 544). However, from this sample, 53 cases had incomplete item‐level data and were excluded from the final analysis (discussed in Statistical Analysis section). There was no difference between the group who were excluded because of questionnaire item missingness and the main sample, based on sex (*p* = .66), age (*p* = .55), placement duration (*p* = .91), placement type (*p* = .16) or ethnicity (*p* = .17).

Common reasons for children declining participation were being too busy, not being interested in the study, and not wanting to be identified with their experiences of care. Children and carers were given the options of completing questionnaires online, by postal pack, in‐person with a researcher or digitally via a video or telephone call.

### Measures

#### Strengths and Difficulties Questionnaire (SDQ)

The SDQ (Goodman, 1997) is an extensively validated 25‐item questionnaire with child‐ and carer‐report versions used to assess child internalising and externalising difficulties. The measure provides a total problems score (20 items) from the sum of four subscales (emotional problems, peer problems, conduct problems, hyperactivity). Each can also be used as a stand‐alone sub‐scale. There is also a prosocial skills subscale, not used in the total score calculation. Whilst there is a newer 4‐category system for identifying level of clinical need (close to average, slightly raised, high, very high), the more established 3‐category system is what is used in children's social care. These clinically validated cut‐offs are labelled normal, borderline, abnormal (Goodman, 1997). Whilst the new 4‐category system removed the negative, stigmatising language of the original 3‐category system, it remains the system used in children's social care so for ease of interpretation this paper uses the language of the 3‐category system. For transparency, the proportions of children who fell into each of the 4‐categories based on carer‐ and child‐report is reported in Table S1 in Supporting Information S2. The measure showed very good internal consistency for child report (*α* = .85) and caregiver report (*α* = .87) as well as for the emotional sub‐scale for child‐ (*α* = .75) and caregiver report (*α* = .74).

#### Child Revised Impact of Events Scale (CRIES‐8)

The CRIES‐8 (Perrin et al., 2005) is a validated self‐report screening tool for PTSD symptomology in children 8–18 years. The CRIES‐8 is designed to be a brief screening tool and covers two core symptoms of PTSD (re‐experiencing and avoidance). Each item is scored on a 4‐point scale, being either 0 (not at all), 1 (rarely), 3 (sometimes), or 5 (often), meaning total scores can range from 0 to 45. In the validation of this measure a score of 17 or above was identified as the best cut‐off for detecting clinically elevated symptoms (Perrin et al., 2005). The measure showed good internal consistency (*α* = .88).

#### Revised Child Anxiety and Depression Scale (RCADS‐25)

The RCADS‐25 (Ebesutani et al., 2017) is a well validated 25‐item questionnaires with child‐ and carer‐report versions, with this study only reporting on child‐report RCADS‐25 questionnaires. Each item is scored on a 4‐point scale of 0 (never), 1 (sometimes), 2 (often), and 3 (always). Items can be summed into depression and anxiety subscales, as well as total scores, with scores being transformed into sex and age adjusted *t*‐scores that are compared to clinical cut‐offs based on normative datasets (Ebesutani et al., 2017). The measure showed good internal consistency (*α* = .94).

### Statistical analyses

Measures were coded using measure‐specific protocols. As these differ by measure, we ultimately only included participants where relevant scale scores could be calculated.

Descriptive statistical analyses were performed to summarise the mental health need of the included sample of children in care including means, standard deviations, and percentages of children scoring above thresholds. We used kappa scores to explore agreement between carer and child‐report, in terms of scores being in the normal, borderline, or abnormal range, interpreted in line with Landis and Koch's (1977) thresholds. We are focused on categories as we are ultimately exploring the sufficiency of the SDQ cut‐offs for detecting other clinically elevated (above threshold) symptom needs.

The main analysis focused on understanding the sufficiency of the SDQ total score as a sole screening tool for children in care. First, as all measure scores were positively skewed, we conducted a square root transformation (Ferketich & Verran, 1994). We then used bivariate correlations to explore the strength of associations between measure total scores (associations with each subscale of the SDQ are in Table S1 in Supporting Information S3), interpreted based on Cohen's (1988) effect size categorisations. As a sensitivity check we also conducted Spearman's rho correlations on the raw data, which showed the same pattern of results, as did sensitivity analyses using untransformed data. Child‐carer SDQ agreement was operationalised as the absolute difference between child and carer total SDQ scores (as a positive integer). The relationship between carer‐child SDQ agreement and placement duration was investigated through bivariate correlations to assess the role of this potential confounder.

Next, using crosstabs we explored the false negative rates, which reflect the proportion of children that scored below threshold on the self‐ or carer‐report SDQ (i.e., would not be flagged as struggling on this measure), who did score above threshold on the PTSD, anxiety, or depression symptom measures. This reflects the number of children ‘missed’ by the SDQ. To be conservative, we used an SDQ cut‐off of below borderline. For these analyses we first explored sufficiency using the total SDQ score cut‐offs, and then repeated analyses using the cut‐offs for the emotional problems scale only (again with the cut‐off of below borderline vs. borderline or abnormal range). This was done as PTSD, anxiety and depression are all defined as internalising disorders characterised primarily by symptoms related to emotional difficulties. This avoids low externalising difficulties potentially masking the SDQ's sensitivity to these condition‐specific symptoms when using total scores alone, in line with approaches from previous community sample screening studies (Armitage et al., 2023). We also explored detection rates for carer‐report SDQ only, child‐report SDQ only, and combined carer‐ and child‐report, to understand which method better detects these disorder‐specific elevated symptom needs.

Finally, we explored whether there were demographic differences in children who were ‘missed’ on the carer‐report SDQ. We focused on carer report SDQ as this is what local authorities use and we wanted to understand whether any demographic groups might be missed by this practice. Independent samples *t*‐tests were used to explore whether missingness (i.e., did the SDQ detect or miss clinically‐elevated symptoms) was associated with child age and placement duration, and chi‐squared tests were use to explore this association with sex, ethnicity, and placement type. Due to limited sample size of minoritized ethnic groups, ethnicity was coded as white versus any other ethnicity. Placement type was coded as ‘family‐style’ or ‘private household’ placement (foster care, kinship care) versus other (residential care, semi‐independent care, and other types of placements outside of a ‘family’ home).

## RESULTS

### Descriptives and child‐carer agreement

Most (65%; *n* = 391) children scored above threshold on at least one self‐report mental health measure. From child self‐report over half (53.2%) of the sample were in the clinically elevated range for PTSD symptoms, 15.9% were in the borderline or clinically elevated range for anxiety, and 14.3% were in the borderline or elevated range for depression symptoms. Child self‐report and carer‐report scores are presented in Table 2.

**TABLE 2:** Description of sample mental health need.

	Young person‐report (*n* = 491)	Carer report (*n* = 342)
	*n* (%)	*n* (%)
SDQ total		
Normal	313 (63.7)	174 (50.9)
Borderline	87 (17.7)	39 (11.4)
Abnormal	91 (18.5)	129 (37.7)
SDQ emotional		
Normal	393 (80.0)	196 (57.3)
Borderline	44 (9.0)	44 (12.9)
Abnormal	54 (11.0)	102 (29.8)
CRIES‐8		
Below threshold	230 (46.8)	‐
Above threshold	261 (53.2)	‐
RCADS‐25		
Total—Normal	409 (83.3)	‐
Total—Borderline	24 (4.9)	‐
Total—Clinical	58 (11.8)	‐
Anxiety—Normal	413 (84.1)	‐
Anxiety—Borderline	22 (4.5)	‐
Anxiety—Clinical	56 (11.4)	‐
Depression—Normal	421 (85.7)	‐
Depression—Borderline	16 (3.3)	‐
Depression—Clinical	54 (11.0)	‐

	*M* (SD)	
SDQ^a^		
Total score	13.57 (6.76)	13.96 (7.69)
Emotional symptoms	3.26 (2.47)	3.16 (2.42)
Peer problems	2.67 (2.02)	3.15 (2.40)
Conduct problems	2.45 (1.99)	2.73 (2.47)
Hyperactivity	5.18 (2.71)	4.92 (2.98)
Prosocial behaviour	7.70 (2.07)	7.00 (2.47)
CRIES‐8^b^		
Total score	17.32 (11.60)	‐
RCADS‐25^c^		
Total *t*‐score	48.54 (18.30)	‐
Anxiety *t*‐score	48.48 (17.24)	‐
Depression *t*‐score	48.58 (16.02)	‐

Abbreviations: CRIES‐8, Child Revised Impact of Events Scale; RCADS‐25, Revised Child Anxiety and Depression Scale; SDQ, Strengths and Difficulties Questionnaire.

^a^ Borderline cut‐off for SDQ total scores is 16 for child‐report and 14 for carer‐report, the Abnormal cut off is 20 for child‐report and 17 for carer‐report.

^b^ Clinical cut off for CRIES‐8 is 17.

^c^ Borderline cut off for RCADS‐25 adjusted *t*‐scores is 65 for and clinical cut off is 70.

For the SDQ total difficulties, from child self‐report 63.7% (*n* = 313) were in the normal range, 17.7% (*n* = 87) were in the borderline range, and 18.5% (*n* = 91) were in the abnormal range. On the emotional subscale rates were: 80.0% normal, 9.0%, borderline, 11.0% abnormal. From carer‐report SDQ total difficulties, 50.9% children (*n* = 174) were in the normal range, 11.4% (*n* = 39) were in the borderline range and 37.7% (*n* = 129) were in the abnormal range. For the carer‐report emotional sub‐scale rates were: 57.3% normal, 12.9% borderline, and 29.8% abnormal.

Means and standard deviations are presented in Table 2. Kappa analyses showed fair‐moderate agreement between self‐ and carer‐report at the borderline SDQ total score cut‐off (*κ* = 0.41 [95% CI: 0.32–0.50]) and fair agreement in ratings for the abnormal cut‐off (*κ* = 0.34 [95% CI: 0.24–0.44]), with wide confidence intervals suggesting cut‐off may not meaningfully impact agreement scores.

### Correlations between SDQ, CRIES‐8 and RCADS‐25

Correlations are presented in Table 3. All measures were significantly correlated, with the SDQ child self‐report total and emotional sub‐scale scores showing moderate correlations with PTSD symptoms and strong correlations with anxiety and depression symptoms. For carer‐report SDQ, there were weak associations between carer‐report SDQ total scores, and child‐reported PTSD, anxiety, and depression symptom scores, but moderate associations with the SDQ emotional sub‐scale.

**TABLE 3:** Correlations between mental‐health questionnaires.

	PTSD symptoms^a^	Anxiety symptoms^b^	Depression symptoms^b^
	*r*_*p*_ (*n* = 491)		
Young person report			
SDQ total	0.362**	0.572**	0.607**
SDQ emotional	0.488**	0.729**	0.687**

	*r*_*p*_ (*n* = 342)		
Carer report			
SDQ total	0.204**	0.172**	0.240**
SDQ emotional	0.313**	0.380**	0.382**

Abbreviations: CRIES‐8, Child Revised Impact of Events Scale; PTSD, posttraumatic stress disorder; RCADS‐25, Revised Child Anxiety and Depression Scale; SDQ, Strengths and Difficulties Questionnaire.

^a^ CRIES‐8 total scores.

^b^ RCADS‐25 anxiety and depression sub‐scale adjusted *t*‐scores.

*Significant at .05 alpha level. **Significant at .01 alpha level.

No significant association was found between the difference in child‐ and carer‐report total SDQ scores and placement duration, suggesting agreement between carer and child‐report SDQ was not associated with how long children had lived with carers.

### Threshold comparisons: SDQ, CRIES‐8 (PTSD) and RCADS‐25 (anxiety, depression)

#### Child SDQ report

Of children who self‐reported PTSD symptoms in the clinically elevated range (*n* = 261), 51.0% did not score in the borderline or abnormally elevated range from the self‐report SDQ total scores. Of children who self‐reported clinically elevated anxiety symptoms (*n* = 78), 28.2% did not score themselves in the borderline or abnormally elevated range on the self‐report SDQ. Similarly, for those in the clinical range for depression symptoms (*n* = 70), 25.7% did not score in the elevated ranges on the self‐report SDQ. That is, the self‐report SDQ borderline cut‐off missed half of children with clinically elevated PTSD symptoms, and over a quarter of children with either clinically elevated anxiety or depression symptoms. These findings are summarised in Table 4.

**TABLE 4:** Crosstab comparing child‐ and carer‐report SDQ categorisations with CRIES‐8 and RCADS‐25 clinical cut offs.

	PTSD symptoms^a^	
	Below clinical threshold	At or above clinical threshold
Child‐report SDQ total score (*n* = 491)		
Below threshold^c^	179 (36.5%)	134 (27.3%)
Above threshold	51 (10.4%)	127 (25.9%)
Carer‐report SDQ total score (*n* = 342)		
Below threshold	90 (26.3%)	84 (24.6%)
Above threshold	79 (23.1%)	89 (26.0%)

	Anxiety symptoms^b^	
	Below clinical threshold	At or above clinical threshold
Child‐report SDQ total score (*n* = 491)		
Below threshold	291 (59.3%)	22 (4.5%)
Above threshold	122 (24.8%)	56 (11.4%)
Carer‐report SDQ total score (*n* = 342)		
Below threshold	154 (45.0%)	20 (5.8%)
Above threshold	143 (41.8%)	25 (7.3%)

	Depression symptoms^b^	
	Below clinical threshold	At or above clinical threshold
Child‐report SDQ total score (*n* = 491)		
Below threshold	295 (60.1%)	18 (3.7%)
Above threshold	126 (25.7%)	52 (10.6%)
Carer‐report SDQ total score (*n* = 342)		
Below threshold	157 (45.9%)	17 (5.0%)
Above threshold	148 (43.3%)	20 (5.8%)

Abbreviations: CRIES‐8, Child Revised Impact of Events Scale; PTSD, posttraumatic stress disorder; RCADS‐25, Revised Child Anxiety and Depression Scale; SDQ, Strengths and Difficulties Questionnaire.

^a^ CRIES‐8 total scores.

^b^ RCADS‐25 anxiety and depression sub‐scale adjusted *t*‐scores.

^c^ Thresholds used are the ‘borderline’ cut‐off scores for the 3‐category SDQ.

#### Carer SDQ report

Of the 342 children who had caregiver data, 173 (50.5%) scored themselves above the clinical threshold for PTSD symptoms. Of these, 48.6% did not score in the borderline or abnormally elevated range on the carer‐report SDQ total score. Of the children who self‐reported clinically elevated anxiety symptoms (*n* = 45), 44.4% did not score in the elevated ranges on the borderline or abnormal range on the carer‐report SDQ, whilst of those with clinically elevated depression symptoms, 46.0% (*n* = 37) were not in the elevated ranges on the carer‐report SDQ. To summarise, the carer‐report SDQ borderline or above cut‐off missed nearly half of children with clinically elevated PTSD, anxiety, and depression symptoms. These findings are summarised in Table 4.

#### Emotional subscale

Analyses were repeated with the emotional sub‐scale of the carer and self‐report SDQ. Reliance on the child self‐report emotional sub‐scale alone led to higher rates of missing elevated PTSD, anxiety, and depression symptom scores. The borderline threshold on the self‐report emotional difficulties subscale missed 68.9% of young people above threshold for PTSD, 33.3% for anxiety, and 38.6% for depression. The borderline threshold on carer‐reported emotional difficulties missed 48.6% of children above threshold for PTSD, 35.6% for anxiety, and 37.8% for depression, reflecting a slight improvement in identification of anxiety and depression symptoms.

#### Combined carer‐child SDQ report

Of the children who self‐reported PTSD symptoms in the clinical range, 37.5% did not score in the borderline or abnormally elevated range on either the self‐ or carer‐report SDQ total scores. Of the children who self‐reported clinically elevated anxiety symptoms, 24.4% did not score in the borderline or abnormally elevated range on either the self‐ or carer‐report SDQ. Finally, of the children who self‐reported clinically elevated depression symptoms, 21.6% did not score in borderline or abnormal range on either the self‐ or carer‐report SDQ. Thus, combined report detected similar rates to child only report for anxiety and depression symptoms detection (i.e., approximately a quarter of children missed with elevated symptoms) but performed better for PTSD detection compared to child only report (i.e., where 50% of elevated PTSD symptoms were missed).

### Differences between children missed and detected by the SDQ

Results showed no consistent pattern between children ‘missed’ or ‘detected’ by carer‐report SDQ by age, sex, ethnicity, placement type or placement duration (for full analyses see Table 5). Having elevated PTSD symptoms missed (but not anxiety or depression) by carer‐report SDQ was significantly associated with being in a longer placement (*p* = .02). Boys' (compared to girls') elevated anxiety symptoms were more likely to be missed by the carer‐report SDQ (*p* = .04) and depression symptoms more likely to be missed by child self‐report (*p* = .03). Having elevated PTSD and anxiety symptoms was more likely to be missed on the self‐report SDQ for non‐white versus white children (*p* = .04), and PTSD symptoms more likely to be missed for those in foster or kinship placement versus other placement types (*p* = .02). However, none of these findings held consistently across measures or reporter‐type, making conclusions difficult.

**TABLE 5:** Differences in demographic characteristics by children scoring above clinical thresholds for PTSD, anxiety and depression symptoms and below SDQ thresholds.

	Carer report^a^								
	SDQ missed versus detected elevated symptoms								
	PTSD symptoms			Depression symptoms			Anxiety symptoms		
	(*n*_miss_ = 84, *n*_detect_ = 89)			(*n*_miss_ = 17, *n*_detect_ = 20)			(*n*_miss_ = 20, *n*_detect_ = 25)		
	*n*_miss_ (%)	*n*_detect_ (%)	*χ* ^2^ (*p*)	*n*_miss_ (%)	*n*_detect_ (%)	*χ* ^2^ (*p*)	*n*_miss_ (%)	*n*_detect_ (%)	*χ* ^2^ (*p*)
Boys	48 (57.1)	39 (43.8)	3.07 (.080)	11 (64.7)	14 (70)	0.19 (.732)	18 (90.0)	16 (64.0)	4.07 (.044)*
Non‐white children	17 (20.2)	17 (19.1)	0.04 (.851)	4 (23.5)	7 (35)	0.58 (.447)	7 (35.0)	7 (28.0)	0.25 (.614)
Carer‐led placement	80 (95.2)	78 (87.6)	3.15 (.076)	15 (88.2)	16 (80)	0.46 (.498)	18 (90.0)	18 (72.0)	2.25 (.134)

	*M*_miss_ (SD)	*M*_detect_ (SD)	*t* (*p*)	*M*_miss_ (SD)	*M*_detect_ (SD)	*t* (*p*)	*M*_miss_ (SD)	*M*_detect_ (SD)	*t* (*p*)
Placement duration	3.80 (3.15)	2.85 (2.13)	−2.29 (.023)*	4.05 (2.56)	3.14 (3.86)	−0.83 (.412)	4.65 (4.18)	3.13 (3.43)	−1.34 (.188)
Child age	13.27 (2.52)	13.13 (2.35)	−0.38 (.708)	14.88 (2.62)	15.50 (1.82)	0.82 (.420)	15.00 (2.35)	14.30 (2.64)	0.93 (.359)

	Child report								
	SDQ missed versus detected elevated symptoms								
	PTSD symptoms			Depression symptoms			Anxiety symptoms		
	(*n*_miss_ = 134, *n*_detect_ = 127)			(*n*_miss_ = 18, *n*_detect_ = 52)			(*n*_miss_ = 22, *n*_detect_ = 56)		
	*n*_miss_ (%)	*n*_detect_ (%)	*χ* ^2^ (*p*)	*n*_miss_ (%)	*n*_detect_ (%)	*χ* ^2^ (*p*)	*n*_miss_ (%)	*n*_detect_ (%)	*χ* ^2^ (*p*)
Boys	72 (53.7)	68 (53.4)	0.001 (.976)	9 (50.0)	40 (76.9)	4.62 (.032)*	17 (77.3)	41 (73.2)	0.14 (.712)
Non‐white children	39 (29.1)	23 (18.1)	4.35 (.037)*	8 (44.4)	11 (21.2)	3.67 (.055)	11 (50.0)	14 (25.0)	4.53 (.033)*
Carer‐led placement	115 (85.8)	94 (74.0)	5.70 (.017)*	14 (77.8)	33 (63.5)	1.24 (.265)	17 (77.3)	38 (67.9)	0.67 (.412)

	*M*_miss_ (SD)	*M*_detect_ (SD)	*t* (*p*)	*M*_miss_ (SD)	*M*_detect_ (SD)	*t* (*p*)	*M*_miss_ (SD)	*M*_detect_ (SD)	*t* (*p*)
Placement duration	3.53 (3.08)	2.99 (2.14)	−1.35 (.180)	4.40 (2.98)	3.18 (3.38)	0.10 (.917)	4.52 (4.56)	3.38 (3.30)	−0.97 (.337)
Child age	13.86 (2.60)	14.17 (2.56)	0.98 (.328)	15.56 (2.38)	15.62 (2.00)	−1.04 (.303)	14.55 (2.79)	15.36 (2.17)	1.23 (.229)

Abbreviations: PTSD, posttraumatic stress disorder; SDQ, Strengths and Difficulties Questionnaire.

^a^ Due to reliance carer report (*n* = 342).

*Significant at .05 significance level. **Significant at .01 significance level.

## DISCUSSION

Our study's findings confirm the high level of mental health need amongst children living in care in the UK (specifically, England and Wales). Almost two‐thirds of the sample scored in the clinical range for at least one measured internalising condition (anxiety, depression, PTSD), in line with previous research with this population (Coughlan et al., 2024; Devaney et al., 2023; Ford et al., 2007). Significant correlations were found between both self‐ and carer‐report SDQ scores and scores on disorder‐specific measures of PTSD, anxiety and depression symptoms. But, when using the established and widely used cut‐offs, around half of children who scored above clinical thresholds for posttraumatic stress, anxiety and depression symptoms were not identified as above clinical threshold on the carer‐report SDQ. There were also only fair to moderate rates of agreement between self‐ and carer‐report SDQ score categorisations, with carers reporting higher scores. Higher detection rates of clinically raised posttraumatic stress, depression and anxiety symptoms were found when combining self‐ and carer‐report SDQs, compared to carer‐only report.

Currently, there is no agreed approach to assessing the mental health of children living in care across the UK. In England the statutory administrative data guidance places reliance on the sole use of the carer‐report SDQ total scores to flag children living in care facing mental health difficulties (Department for Education, 2024b). For many children in care, this is the only standardised mental health measure completed on their needs. The findings of our study show that the sole use of the carer‐report SDQ total score risks missing large numbers of children in care experiencing trauma‐related and common mental health symptomology—in this case, anxiety, depression and posttraumatic stress. This builds on existing research which has criticised the exclusive use of the SDQ as liable to miss certain profiles and nuances in the mental health needs of children living in care (Frogley et al., 2019; Tarren‐Sweeney et al., 2019; Wright et al., 2019). Here, we have shown the proportion of children with clinically‐elevated anxiety, depression and PTSD symptoms likely missed by current screening practice. The statutory use of the SDQ in England provides an important yearly snapshot of the mental health of children in care—indeed, the addition of mandatory SDQ reporting would be an improvement on the lack of any statutory mental health screening for children living in care in Wales, Scotland and Northern Ireland. However, many carers and social care professionals in England have expressed frustration that it is a data gathering exercise with no benefits to the young people (Frogley et al., 2019). In part, this is because of poor understanding of the purpose of the SDQ—it is not designed to capture all mental health difficulties, although that is how it is often used. While there have been attempts to enhance mental health assessments for children in care, these have faced challenges of poor uptake and sustainability (Brown et al., 2021). There is growing evidence that common mental health conditions remain under‐detected in children in care, despite clear evidence of their high prevalence (Ford et al., 2007). The lack of use of condition‐specific mental health screening tools may exacerbate this problem, with potential implications for access to services (McGuire et al., 2022). Concerningly, the sole use of the SDQ is increasingly being relied on by NHS CAMHS to make triaging decisions (Johnston & Gowers, 2005; Mathai et al., 2002). Thus, findings from the current study also serve as a warning for these systems also.

Our analysis shows only moderate‐fair agreement between carer‐ and self‐report SDQ total scores, similar to other research (McSherry et al., 2019). From mean scores, children generally rated symptoms lower than their carer. This is not a problem per se, as there is extensive research that parents and children have limited agreement on mental health measures of internalising difficulties (Cleridou et al., 2017). For a child in care this may be exacerbated further by the carer being a newer person in that child's life. Nevertheless, compared to carer‐report only, the combined review of self‐ and carer‐report SDQ total scores led to higher detection rates of children with elevated self‐report posttraumatic stress, anxiety and depression scores (although many children were still missed). The developers of the SDQ suggest triangulation between child, carer and professional report (Bergström & Baviskar, 2021; Goodman, 1997), but this is not how the SDQ is commonly used in children's social care in the UK and there would likely be major feasibility issues in adopting this as standard practice. Our findings highlight the importance of giving children the opportunity to give their perspectives on their own mental health, for example, as part of annual health reviews, to give more opportunities for timely intervention. Whilst professionals often express concern about the use of condition‐specific screening tools (McGuire et al., 2025), large primary research studies with children living in care consistently show that these types of measures are widely acceptable to young people in care (e.g., Carter et al., 2025). Such evidence is relevant for both children's social care and CAMHS.

### Limitations

This study has explored sufficiency of the carer‐report SDQ against standardised symptom screening tools with validated cut‐offs but has not used full diagnostic assessments. Our focus here on screening tools was intentional, as this mirrors current social care and mental health care practice in England, where carer‐report SDQ is largely the sole screening tool used by local authorities, and where in mental health services it would be unusual to use full diagnostic instruments, and there is a culture of moving away from diagnostic language (Callaghan et al., 2003; McGuire et al., 2025). Thus, whilst this might be a methodological limitation (in that we cannot determine whether or not the child meets diagnosis for PTSD, anxiety or depression), the focus on symptom severity and screening tools increases the real‐world validity of the findings. Our findings suggest child‐report SDQ is best at detecting child‐report disorder‐specific symptoms, unsurprisingly given single‐informant bias. Yet, past work has shown that carer‐report SDQ is the most accurate at identifying the presence or absence of a full diagnosis based on a professional clinical assessment, although this paper did not detail who was ‘missed’ (Goodman et al., 2003).

Second, while we used established clinical symptom cut‐offs, these cut‐offs are all validated in samples of children not in care. There is no evidence on whether different cut‐offs may be needed for children in care. For the PTSD symptom tool in particular, there were high rates of clinically‐elevated symptomology, which may indicate the measure is also capturing broader general distress. That said, a previous study showed, in a sample of 141 children in care in Northern Ireland, a high proportion of children scoring above clinical thresholds on disorder‐specific screening tools (e.g., CRIES‐8) fit clinician‐rated criteria for internalising diagnoses (e.g., PTSD) (Devaney et al., 2023). All screening tools used here are also part of the NHS National Clinical Content Repository tools and measures library.

Third, our study was limited by missing carer‐data for a proportion of the wider sample of children in care, with older children in residential group homes less likely to have carer‐report. This is a difficult reflection of the lives of many older children in care, who do not perceive they have a safe and supportive adult in their life.

## CONCLUSION

The SDQ remains an important tool for providing a snapshot of the mental health needs of children in care, which could usefully inform the development of service provision. However, social care and mental health services should recognise that relying on this tool alone, risks missing large numbers of children in care with high symptoms of common and trauma‐related mental health conditions, with potential implications for referrals and access to evidence‐based support.

## AUTHOR CONTRIBUTIONS

**Eva A. Sprecher**: Conceptualization; data curation; formal analysis; investigation; methodology; project administration; writing—original draft; writing—review and editing. **Katherine Shelton**: Conceptualization; formal analysis; funding acquisition; investigation; methodology; supervision; writing—original draft; writing—review and editing. **Lisa Holmes**: Conceptualization; formal analysis; funding acquisition; investigation; methodology; project administration; supervision; writing—original draft; writing—review and editing. **Bethan Carter**: Conceptualization; data curation; formal analysis; investigation; methodology; project administration; writing—original draft; writing—review and editing. **Charlotte Robinson**: Data curation; investigation; methodology; project administration; writing—original draft; writing—review and editing. **Maryam Javed**: Conceptualization; data curation; investigation; project administration; writing—review and editing. **Ruby Khan**: Formal analysis; data curation; methodology; writing—original draft. **John MacLeod**: Conceptualization; funding acquisition; investigation; methodology; writing—review and editing. **Jeongeun Park**: Conceptualization; formal analysis; methodology; writing—original draft; writing—review and editing. **Julie Selwyn**: Conceptualization; funding acquisition; methodology; supervision; writing—original draft; writing—review and editing. **Iram Siraj**: Funding acquisition; supervision; investigation; writing—review and editing. **Ching So**: Formalanalysis; data curation; methodology;writing—original draft. **Rachel M. Hiller**: Conceptualization; formal analysis; investigation; methodology; writing—original draft; writing—review and editing.

## CONFLICT OF INTEREST STATEMENT

The authors declare no conflicts of interest.

## ETHICAL CONSIDERATIONS

All procedures involving human participants were approved by University of Bath (Ref 16/IEC08/0025) and UCL (Ref 22253/001) Research Ethics Committees. Local approval was gained from participating local authorities and national approval was gained for the ReThink Programme (due to project scale) from the Association of Directors of Children's Services. Written consent was provided by the local authority (who hold parental responsibility), with assent provided by the child (or consent if 16+ years old). Caregivers provided their own written consent.

## Supporting information


Supporting Information S1

## Data Availability

Analytic code, data and research materials are available on request to the corresponding author. Data presented here are, in part, baseline data from an ongoing longitudinal project (Preregistered: https://osf.io/7qx54) and will be available via UK Data Service at the conclusion of the full project.
