# Supplementary material for: Sufficiency of current practice: How well does the Strengths and Difficulties Questionnaire detect clinically elevated posttraumatic stress, anxiety, and depression symptoms in children in care?
Source: JCPP Adv. 2025 Oct 30;6(2):e70058. doi: 10.1002/jcv2.70058 (PMC13260667; doi:10.1002/jcv2.70058)
Supplement: Supplementary file 1 — Supporting Information S1 [file JCV2-6-e70058-s001.docx]

**Sufficiency of current practice: How well does the Strengths and Difficulties Questionnaire detect clinically elevated PTSD, anxiety, and depression symptoms in children in care?**

**Supporting Information**

**Table S1**

Demographic information for sub-sample of child sample where carer-data was available.

|  | **Caregiver and child data available (n = 342)** |
| --- | --- |
|  | M (SD) |
| *Child age* | 13.11 (2.32) |
| *Carer age* | 52.34 (10.02) |
| *Child age at placement start* | 9.17 (3.48) |
| *Placement length* | 3.94 (3.22) |
|  | N (%) |
| *Child Gender* Boy | 171 (50.0) |
| Girl | 160 (46.8) |
| Non-binary | 4 (1.2) |
| Prefer not to say | 3 (0.9) |
| Not known | 4 (1.2) |
| *Child sex* Female | 167 (48.8) |
| Male | 175 (51.2) |
| *Child ethnicity* Asian | 6 (1.8) |
| Black | 20 (5.8) |
| Mixed | 38 (11.1) |
| Other | 6 (1.8) |
| White | 267 (78.1) |
| Not known | 5 (1.5) |
| *Placement type* Foster care | 276 (80.7) |
| Kinship care | 42 (12.3) |
| Residential / semi-independent / supported | 21 (6.1) |
| Other | 1 (0.3) |
| Not known | 2 (0.6) |

**Table S2**

*Description of sample mental health need using 4-category SDQ coding system.*

|  | **4-Category SDQ Coding** |  |
| --- | --- | --- |
|  | **Carer-report (n = 342)** | **Child Self-Report (n = 491)** |
|  | n (%) | n (%) |
| SDQ total^a^ |  |  |
| *Close to average* | 174 (50.9) | 282 (57.4) |
| *Slightly raised* | 39 (11.4) | 81 (16.5) |
| *High* | 41 (12.0) | 35 (7.1) |
| *Very high* | 88 (25.7) | 91 (18.5) |
| *SDQ emotional^b^* |  |  |
| *Close to average* | 196 (57.3) | 338 (68.8) |
| *Slightly raised* | 44 (12.9) | 55 (11.2) |
| *High* | 70 (20.5) | 44 (9.0) |
| *Very high* | 32 (9.4) | 54 (11.0) |

^a^ For carer-report the Close to Average cut-off for SDQ total scores is 14, the High cut-off is 17, and the Very High cut-off is 20. For child self-report this is 15 (Close to Average), 18 (High) and 20 (Very High).

^a^ For carer-report the Close to Average cut-off for SDQ emotional sub-scale scores is 4, the High cut-off is 5, and the Very High cut-off is 7. For child self-report this is 5 (Close to Average), 6 (High) and 7 (Very High).

**Table S3**

Correlations between SDQ sub-scales and PTSD, anxiety and depression symptoms

|  | **PTSD symptoms ^a^** | **Anxiety symptoms ^b^** | **Depression symptoms ^b^** |
| --- | --- | --- | --- |
|  | *r_p_ (*n = 491) | | |
| Young person report |  |  |  |
| *SDQ peer problems* | .229** | .354** | .382** |
| *SDQ conduct problems* | .138** | .267** | .338** |
| *SDQ hyperactivity* | .186** | .303** | .355** |
| *SDQ prosocial* | .066 | .027 | -.118** |
|  | *r_p_ (*n = 342) | | |
| Carer report |  |  |  |
| *SDQ peer problems* | .123* | .090 | .161* |
| *SDQ conduct problems* | .119* | .044 | .177** |
| *SDQ hyperactivity* | .076 | .025 | .032 |
| *SDQ prosocial* | -.085 | -.018 | -.148** |

* significant at .05 alpha level ** significant at .01 alpha level

^a^ CRIES-8 total scores

^b^ RCADS-25 anxiety and depression sub-scale adjusted t-scores

**Table S4**

Crosstab comparing child- and carer-report SDQ emotional sub-scale categorisations with CRIES-8 and RCADS-25 clinical cut offs.

|  |  | **PTSD symptoms ^a^** | |
| --- | --- | --- | --- |
|  |  | *Below clinical threshold* | *At or above clinical threshold* |
| **Child-report SDQ emotional sub-scale (n = 491)** | *Below threshold^c^* | 213 (43.4%) | 180 (36.7%) |
|  | *Above threshold* | 17 (3.5%) | 81 (16.5%) |
| **Carer-report SDQ emotional sub-scale (n = 342)** | *Below threshold* | 112 (32.7%) | 84 (24.6%) |
|  | *Above threshold* | 57 (16.7%) | 89 (26.0%) |
|  |  | **Anxiety symptoms ^b^** | |
|  |  | *Below clinical threshold* | *At or above clinical threshold* |
| **Child-report SDQ emotional sub-scale (n = 491)** | *Below threshold* | 367 (74.7%) | 26 (5.3%) |
|  | *Above threshold* | 46 (9.4%) | 52 (10.6%) |
| **Carer-report SDQ emotional sub-scale (n = 342)** | *Below threshold* | 180 (52.6%) | 16 (4.7%) |
|  | *Above threshold* | 117 (34.2%) | 29 (8.5%) |
|  |  | **Depression symptoms ^b^** | |
|  |  | *Below clinical threshold* | *At or above clinical threshold* |
| **Child-report SDQ emotional sub-scale (n = 491)** | *Below threshold* | 366 (74.5%) | 27 (5.5%) |
|  | *Above threshold* | 55 (11.2%) | 43 (8.8%) |
| **Carer-report SDQ emotional sub-scale (n = 342)** | *Below threshold* | 182 (53.2%) | 14 (4.1%) |
|  | *Above threshold* | 123 (36.0%) | 23 (6.7%) |

^a^ CRIES-8 total scores

^b^ RCADS-25 anxiety and depression sub-scale adjusted t-scores

^c^ thresholds used are the ‘borderline’ cut-off scores for the 3-category SDQ
